# Quantification of P-glycoprotein function at the human blood-brain barrier using [^18^F]MC225 and PET

**DOI:** 10.1007/s00259-023-06363-5

**Published:** 2023-08-08

**Authors:** Pascalle Mossel, Wejdan M. Arif, Giordana Salvi De Souza, Lara Garcia Varela, Chris W. J. van der Weijden, Hendrikus H. Boersma, Antoon T. M. Willemsen, Ronald Boellaard, Philip H. Elsinga, Ronald J. H. Borra, Rudi A. J. O. Dierckx, Adriaan A. Lammertsma, Anna L. Bartels, Gert Luurtsema

**Affiliations:** 1 Department of Nuclear Medicine and Molecular Imaging, University of Groningen, University Medical Center Groningen, Groningen, The Netherlands; 2 College of Applied Medical Science, Department of Radiological Sciences, King Saud University, Riyadh, Saudi Arabia; 3 Molecular Imaging Biomarkers Group, Center for Research in Molecular Medicine and Chronic Diseases (CIMUS), University of Santiago de Compostela (USC), 15706 Santiago de Compostela, Spain; 4 Nuclear Medicine Department and Molecular Imaging Group, Health Research Institute of Santiago de Compostela (IDIS), SERGAS, 15706 Santiago de Compostela, Spain; 5 Department of Radiology, University of Groningen, University Medical Center Groningen, Groningen, The Netherlands; 6 Department of Radiology and Nuclear Medicine, UMC, Location VUmc, Amsterdam, The Netherlands; 7 Department of Neurology, Ommelander Ziekenhuis Groep, Scheemda, The Netherlands

**Keywords:** P-glycoprotein, ABC-transporter, Pharmacokinetic modelling, Test-retest, Neuro-imaging

## Abstract

**Introduction:**

P-glycoprotein (P-gp) is one of the most studied efflux transporters at the blood-brain barrier. It plays an important role in brain homeostasis by protecting the brain from a variety of endogenous and exogeneous substances. Changes in P-gp function are associated both with the onset of neuropsychiatric diseases, including Alzheimer’s disease and Parkinson’s disease, and with drug-resistance, for example in treatment-resistant depression. The most widely used approach to measure P-gp function in vivo is *(R)-*[^11^C]verapamil PET. *(R)-*[^11^C]verapamil is, however, an avid P-gp substrate, which complicates the use of this tracer to measure an increase in P-gp function as its baseline uptake is already very low. [^18^F]MC225 was developed to measure both increases and decreases in P-gp function.

**Aim:**

The aim of this study was (1) to identify the pharmacokinetic model that best describes [^18^F]MC225 kinetics in the human brain and (2) to determine test-retest variability.

**Methods:**

Five (2 male, 3 female) of fourteen healthy subjects (8 male, 6 female, age 67 ± 5 years) were scanned twice (injected dose 201 ± 47 MBq) with a minimum interval of 2 weeks between scans. Each scanning session consisted of a 60-min dynamic [^18^F]MC225 scan with continuous arterial sampling. Whole brain grey matter data were fitted to a single tissue compartment model, and to reversible and irreversible two tissue-compartment models to obtain various outcome parameters (in particular the volume of distribution (*V*_*T*_), *K*_*i*_, and the rate constants *K*_1_ and *k*_2_). In addition, a reversible two-tissue compartment model with fixed *k*_3_/*k*_4_ was included. The preferred model was selected based on the weighted Akaike Information Criterion (AIC) score. Test-retest variability (TRTV) was determined to assess reproducibility.

**Results:**

Sixty minutes post-injection, the parent fraction was 63.8 ± 4.0%. The reversible two tissue compartment model corrected for plasma metabolites with an estimated blood volume (*V*_*B*_) showed the highest AIC weight score of 34.3 ± 17.6%. The TRVT of the *V*_*T*_ for [^18^F]MC225 PET scans was 28.3 ± 20.4% for the whole brain grey matter region using this preferred model.

**Conclusion:**

[^18^F]MC225 *V*_*T*_, derived using a reversible two-tissue compartment model, is the preferred parameter to describe P-gp function in the human BBB. This outcome parameter has an average test-retest variability of 28%.

**Trial registration:**

EudraCT 2020-001564-28. Registered 25 May 2020.

**Supplementary Information:**

The online version contains supplementary material available at 10.1007/s00259-023-06363-5.

## Introduction

The blood-brain barrier (BBB) is a physical dynamic boundary between the capillaries of the brain vasculature and brain tissue that is composed of endothelial cells connected by tight junctions. The architecture of the BBB prevents paracellular diffusion of hydrophilic compounds and only small molecules can cross by passive transmembrane diffusion. Transport across the BBB is possible due to the presence of multiple transporters, which regulate the influx and efflux of both exogenous and endogenous compounds across the BBB [1]. P-glycoprotein (P-gp) or ABCB1 is one of those transporters and is responsible for the transport of a variety of compounds, like neurotoxins and drugs. It is an efflux transporter and member of the ATP Binding Cassette (ABC)-transporter family and it plays an important role in maintaining brain homeostasis and protection of the brain through efflux of harmful substances [2]. P-gp expression and function can be altered due to several factors, including administration of pharmaceuticals, stress, ageing, diet, inflammatory responses, and genetic differences [3, 4].

Changes in P-gp function are proposed as possible aetiology of several neurological and psychiatric disorders, including Alzheimer’s disease and Parkinson’s disease [5–8]. In Alzheimer’s disease, decreased P-gp function is associated with a reduced efflux of neurotoxic amyloid, which itself is a P-gp substrate. This causes an imbalance between production and clearance of amyloid, leading to higher levels of neurotoxic amyloid in the brain [9]. On the other hand, several pharmaceuticals are classified as P-gp substrates and the efflux of those therapeutic drugs across the BBB is a potential mechanism of drug-resistance and drug-drug interactions. In treatment-resistant depression, schizophrenia, epilepsy, and oncology, an increase in P-gp function is associated with low bio-availability of pharmaceuticals in the brain [10, 11].

PET imaging is an established method to measure P-gp transporter function in vivo and, as such, it allows for a better understanding of transporter-related pathophysiological processes. Moreover, PET imaging of P-gp transporter function may be useful in selecting the best treatment option for patients with an altered P-gp function in case of drug resistance, identifying patients who are at risk for developing a disease, and evaluating new potential treatment strategies, including P-gp inducer therapy [12–15]. Several P-gp substrates have been labelled in order to image P-gp function at the BBB. These compounds are transported out of the brain by P-gp and therefore P-gp function can be assessed indirectly by measuring uptake of the tracer in the brain. At present, the P-gp substrate *(R)*-[^11^C]verapamil is the most widely used PET tracer to measure P-gp function in a variety of preclinical and clinical studies [16]. *(R)*-[^11^C]verapamil shows high selectivity for the P-gp transporter and, using this tracer, a decrease in P-gp function of patients with Alzheimer’s disease compared with healthy controls has been demonstrated [17]. However, one of the major drawbacks of *(R)*-[^11^C]verapamil is the fact that is a so-called avid or strong substrate. This means that its brain uptake in a normal or healthy P-gp state is very low and therefore reliable quantification is very challenging [18].

To overcome this challenge, Savolainen et al. developed 5-(1-(2-[18F]fluoroethoxy))-[3-(6,7-dimethoxy-3,4-dihydro-1H-isoquinolin-2-yl)-propyl]-5,6,7,8-tetrahydronaphthalen ([^18^F]MC225), a novel PET tracer for measuring P-gp function at the BBB [19]. Pharmacokinetic evaluation showed that [^18^F]MC225 acts as a weak P-gp substrate and that it is selective for P-gp, having no affinity for the BCRP transporter at the BBB as well [20]. In a head-to-head comparison performed in non-human primates (NHP), Garcia Varela et al. showed that brain uptake of [^18^F]MC225 at P-gp baseline levels is higher than that of [^11^C]verapamil, making this tracer better suitable for measuring both increases and decreases in P-gp function [18]. In the first-in-human study of [^18^F]MC225, Toyohara et al. evaluated the tracer in three healthy volunteers and concluded that [^18^F]MC225 has an acceptable radiation dose together with pharmacological safety at the dose that is required for PET imaging. The volume of distribution (*V*_*T*_), used as indicator of P-gp function, was compared with previous P-gp PET tracers, and it was shown that baseline uptake of [^18^F]MC225 was indeed higher than that of any other existing P-gp PET tracer [21]. However, test-retest variability of [^18^F]MC225 has not been studied yet, nor have adequate studies been performed to identify the best kinetic model for quantifying [^18^F]MC225 data.

The aim of the present study was twofold: First, [^18^F]MC225 behaviour in healthy volunteers was analysed to identify the pharmacokinetic model that best describes the kinetic behaviour of this novel P-gp PET tracer in the brain based on accuracy, precision, and biological assumptions. Second, the selected model was used to evaluate test-retest variability of [^18^F]MC225.

## Experimental design

### Study population

Fourteen healthy volunteers, 8 male and 6 female (mean age 67 ± 5 years), participated in the study. Five of the subjects (2 male, 3 female) underwent a test-retest protocol, the other nine only received a baseline scan. Subjects were free of any medication affecting P-gp function and had no history of any neurological, psychiatric or psychological disease. As a safety precaution, for each subject, one blood sample was obtained 2 weeks before the first scan to evaluate kidney function (eGFR). A Mini Mental State Examination (MMSE) and a neurological examination were performed prior to the PET scans to exclude any neurological disease that would affect P-gp function. In addition, an MRI scan was used to exclude any cerebral disease.

Written informed consent was obtained from all study participants. The study was approved by the Medical Ethics Review Committee of the University Medical Center Groningen, protocol ID 201900414, NCT 202100647.

### Data acquisition

Following [^18^F]MC225 (200 MBq) administration, each subject underwent a 60-min dynamic PET scan with continuous arterial blood sampling. [^18^F]MC225 was administered as a continuous injection over 60 s via the antecubital vein. All PET scans were acquired using a Siemens Biograph Vision PET/CT System (Siemens Healthcare GmbH, Erlangen, Germany). A low-dose CT was performed for attenuation correction. There was a minimum interval of 2 weeks between test and retest [^18^F]MC225 scans to avoid any side effects of the arterial sampling procedure.

A sagittal 3D T1w MPRAGE (TR: 2300 ms; TE: 2.31 ms; TI: 900 ms; flip angle: 8°; slice thickness: 0.9 mm; voxel size: 0.9 × 0.9 × 0.9 mm) MRI scan was obtained on the same day as the PET scan, as anatomical reference for the PET scans. MRI scans were performed using a 3.0 T Siemens Magnetom Prisma (Siemens Healthcare GmbH, Erlangen, Germany) with a 64-channel head coil.

### Radiochemistry

[^18^F]MC225 was manufactured at the department of Nuclear Medicine and Molecular Imaging of the University Medical Center Groningen (EU-GMP production licence: 108964 F), as described previously [20] For this human study, the tracer was implemented and validated in the GMP facility using an Eckert and Ziegler Modular-Lab PharmTracerSynthesis Module. The molar activity was  > 25000 GBq/mmol, and the radiochemical purity was  > 98% in all cases.

### Arterial blood sampling

An arterial cannula was placed in the radial artery and continuous arterial blood sampling was performed during the [^18^F]MC225 PET scans. The blood monitor (Twilite Sampling System, Swisstrace, Switzerland) was started 30 min prior to the scan without blood running through the system to enable a background correction. Then, 30 s before the start of the scan, the arterial cannula was connected to the blood monitor and blood measurements were started. In addition, five manual samples were taken at 5, 10, 20, 40, and 60 min after the start of the scan for calibration of the whole blood curve and to correct the plasma input curve for radiolabelled metabolites.

### Metabolite analysis of [^18^F]MC225 in arterial plasma

To obtain the metabolite corrected plasma input function of [^18^F]MC225, the manual blood samples were used to measure both the plasma-to-whole-blood ratio and the radioactive fraction of [^18^F]MC225 in plasma, the latter using thin layer chromatography (TLC, F-254 silica plates, Sigma-Aldrich, Germany). Protein precipitation of the plasma samples was performed by addition of 50 µL acetonitrile. The samples were centrifuged for 5 min at 3700 rate per minute (rpm). Protein free plasma samples with a volume of 20 μL were then applied to a TLC plate and placed in eluent (10% MeOH, 90% EtOAc). After elution, radioactivity of the TLC plates was measured using a biomolecular imager (Amersham Typhoon, Global Life Sciences Solutions, USA) and the ratio between parent fraction and radioactive metabolites was calculated (OptiQuant 03.00, Image Analysis Software PerkinElmer, Groningen, The Netherlands).

## Data analysis

### Image reconstruction

No pre-processing smoothing or filter was applied for the [^18^F]MC225 PET scan. List mode data from the emission scan (8 iterations, 5 subsets) were reconstructed into 26 frames (1 × 10, 10 × 5, 1 × 10, 2 × 30, 3 × 60, 2 × 150, 4 × 300, 3 × 600 s) with a voxel size of 0.9 × 0.9 × 0.9. PET images were corrected for attenuation, randoms, dead-time, scatter, decay, and attenuation. MRI images were reconstructed with voxel size of 0.9 × 0.9 × 0.9 mm.

### Plasma input function

The whole blood curve was converted to a plasma curve based on the plasma/whole blood ratios of the 5 manual samples. The metabolite fraction of the 5 manual samples was fitted to a Hill function. The metabolite corrected plasma input curve was obtained by multiplying the total plasma curve with this Hill function. Both the metabolite corrected plasma curve and the whole blood TAC were used as input functions for pharmacokinetic modelling.

### Co-registration

PET images were co-registered to the individual anatomical T_1_ weighted MRI images and spatially normalized to the Montreal Neurological Institute (MNI) space using PMOD (PMOD version 4.0, PMOD Technologies Ltd, Zürich, Switzerland) after motion correction. Motion correction was performed by using the first 12 frames as reference. The co-registered MR was segmented into grey matter, white matter, and extra-cerebral fluid. Nineteen brain regions of interest were defined based on the Hammer’s maximum probability atlas (Hammers N3083), consisting of amygdala, basal ganglia, brainstem, caudate nucleus, cerebellum, cingulate gyrus, corpus callosum, hippocampus, insula, occipital cortex, orbitofrontal cortex, globus pallidus, parietal cortex, putamen, substantia nigra, temporal cortex, thalamus, and white matter. In addition, a whole brain grey matter region was generated by combining all of the previously mentioned grey matter regions. Next, tissue TACs were derived for these preselected brain regions.

### Pharmacokinetic analysis

First, weighting factors based on frame times and decay were applied on the tissue TACs. Then, tissue TACs were fitted to single tissue (1T2k) and both irreversible (2T3k) and reversible (2T4k) two-tissue compartment models using the metabolite corrected plasma and uncorrected whole blood curves as input functions, where the latter was used to estimate fractional blood volume (*V*_*B*_). In addition, TACs were fitted to a reversible two tissue compartment model with a fixed *k*_3_/*k*_4_ parameter, based on the average *k*_3_ (0.18 ± 0.09) and *k*_4_ (0.05 ± 0.01) of the baseline scans of the included subjects. PMOD v4.0 software was used for tracer kinetic modelling. Blood delay was determined by estimating the delay for the whole brain grey matter region based on the best fit to the one tissue compartment model using the first minute of data. This value was then fixed for the other smaller brain regions.

Blood volume was either set to a fixed value (i.e. 5% for grey matter regions and 3% for white matter regions) or estimated fractional blood volume. These fits resulted in best estimates of rate constants (*K*_1_–*k*_4_) and various macroparameters, such as volume of distribution (*V*_*T*_) and net rate of influx (*K*_*i*_), calculated from the microparameters and depending on the actual model being used. The Akaike Information Criteria (AIC) scores were used to identify the best model for each TAC and converted to Akaike weights that can be interpreted as weight of evidence in favour of one of the models in the set [22]. Then, based on the precision of the parameters reflected by the lowest standard error, one parameter was selected as parameter of choice for quantification of the P-gp function.

To describe the outcome parameters, the consensus nomenclature for in vivo imaging of reversibly binding radioligands was used [23].

### Statistical analysis

Descriptive data are described as mean ± standard deviation (SD) unless otherwise specified. Reproducibility was assessed using the test-retest variability (TRTV) of *V*_*T*_, *K*_*I*_, *K*_1_, and *k*_2_, which was calculated for all models as the absolute difference between the results of test and retest outcome parameters, divided by the mean of these values. Differences between test and retest scans were analysed using a paired *t*-test. The statistical threshold for significance was set at 0.05. Statistical analysis was performed using IBM SPSS Statistics version 23 (IBM Corp. Released 2015. IBM SPSS Statistics for Windows, Version 23.0. Armonk, NY).

## Results

### Administration of [^18^F]MC225

Administration of 215 ± 56 MBq [^18^F]MC225 (Supplementary data S2) was well tolerated by all subjects and no complaints or symptoms could be attributed to tracer injection. No significant difference in administered dose, specific activity, or the administered cold compound was found between the test and retest scans.

### Plasma metabolites

**Fig. 1:**
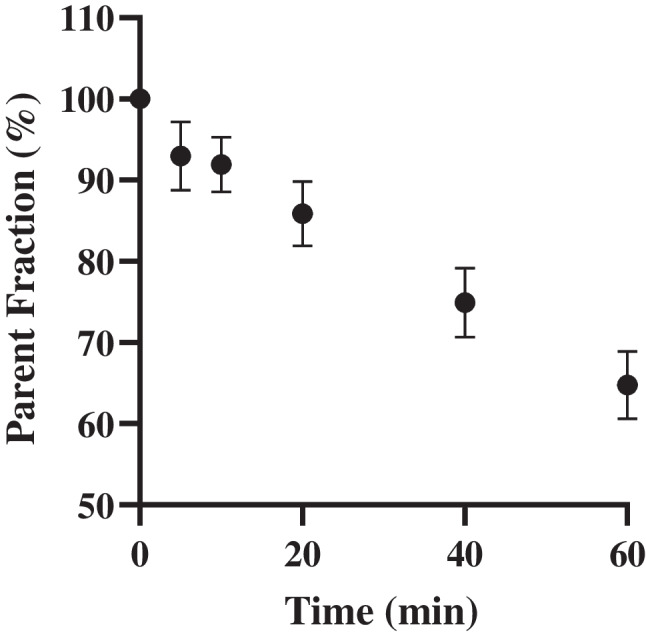
Parent fraction

Sixty minutes post-injection, the parent fraction was 63.8 ± 4.0% (Fig. 1). No significant difference in metabolism between the test (65.2 ± 4.3%) and retest (62.4 ± 3.3%) scans was present (*p* = 0.117).

### Brain uptake of [^18^F]MC225

**Fig. 2:**
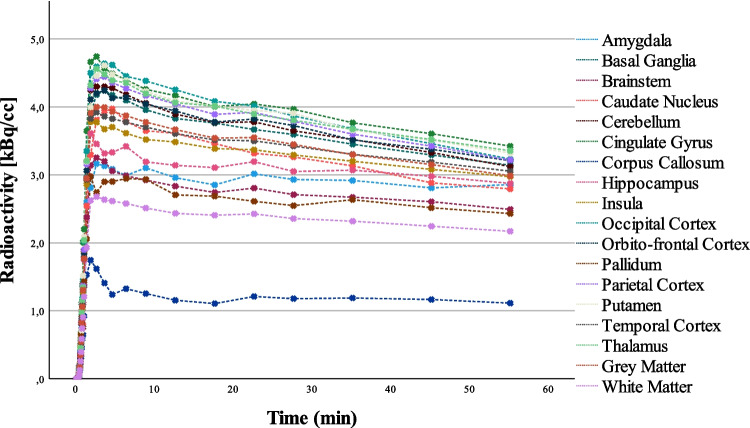
Representative TACs of preselected brain regions in kBq/cc

A representative example of TACs for preselected brain regions is shown in Fig. 2. A clear difference between grey and white matter regions can be seen.

### Model selection

**Fig. 3:**
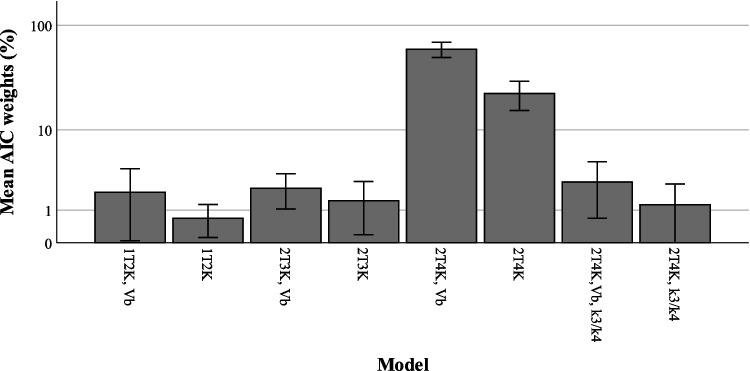
Mean AIC weights

**Fig. 4:**
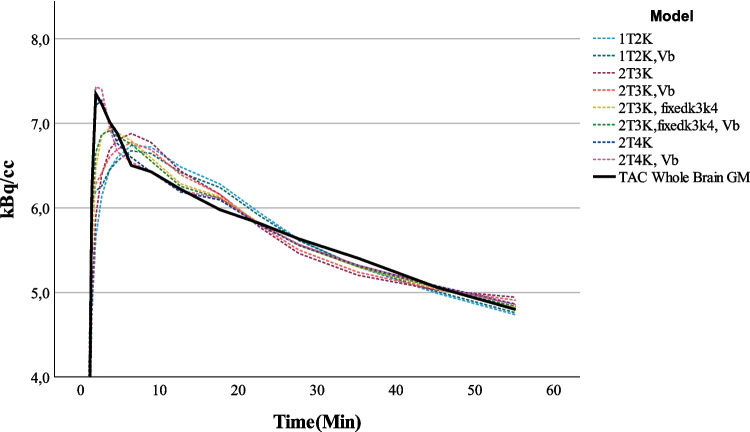
Visual fit of multiple models to the TAC of the whole brain grey matter region

Both Akaike weight scores and visual inspection showed a preference for the reversible two tissue compartment model. Mean Akaike weight scores for the models are shown in Fig. 3. Overall, for a 60-min scan duration, the reversible 2T4K both with an estimated *V*_*B*_ parameter shows the best visual fit and the highest AIC scores of 34 ± 18%. Figure 4 shows an example of the one tissue compartment model and both reversible and irreversible two tissue compartment models, with an estimated *V*_*B*_ as well as a fixed *V*_*B*_. For the reversible two tissue compartment model that was selected as model of choice, *V*_*T*_ showed the lowest standard error of 2.59 ± 3.12%. Both *K*_1_ and *k*_2_ showed higher standard errors of 4.73 ± 3.20 and 24.0 ± 33.4%, respectively. The 2T4K model provided for multiple other brain regions besides the whole brain region good fits (S4).

### Regional analysis of tracer distribution

Table 1 shows *K*_1_, *k*_2_, *k*_3_, *k*_4_, *V*_*T*_, and *V*_*B*_ of [^18^F]MC225 in preselected brain regions of the baseline scans as obtained using a reversible two tissue compartment model. For the baseline scans, the highest *V*_*T*_, *K*_1_, and *k*_2_ values were found in the cingulate gyrus (*V*_*T*_ = 7.64 ± 1.63, *K*_1_ = 0.33 ± 0.20 mL cm^−3^ min^−1^ and *k*_2_ = 0.28 ± 0.13 min^−1^) and thalamus (*V*_*T*_ = 7.04 ± 1.45, *K*_1_ = 0.29 ± 0.13 mL cm^−3^ min^−1^ and *k*_2_ = 0.24 ± 0.21 min^−1^). The lowest *V*_*T*_ was seen in the corpus callosum and white matter, i.e. 3.78 ± 1.14 and 4.93 ± 0.95, respectively. The average *V*_*T*_ was 6.59 ± 1.26 for the whole brain grey matter region and 4.93 ± 0.95 for the whole brain white matter region.

**Table 1:** Kinetic analysis results of regional [^18^F]MC225 data

*Region*	*K*_*1*_* (mL cm*^*−3*^* min*^*−1*^*)*	*k*_*2*_* (min*^*−1*^*)*	*k*_*3*_* (min*^*−1*^*)*	*k*_*4*_* (min*^*−1*^*)*	*V*_*T*_* (mL cm*^*−3*^*)*	*V*_*B*_
*Amygdala*	0.18 ± 0.08	0.16 ± 0.08	0.16 ± 0.07	0.03 ± 0.01	6.56 ± 1.56	0.08 ± 0.06
*Basal ganglia*	0.26 ± 0.10	0.18 ± 0.09	0.17 ± 0.08	0.05 ± 0.01	6.48 ± 1.33	0.09 ± 0.07
*Brainstem*	0.21 ± 0.09	0.20 ± 0.08	0.17 ± 0.07	0.04 ± 0.01	5.47 ± 1.06	0.09 ± 0.08
*Caudate nucleus*	0.26 ± 0.12	0.20 ± 0.14	0.18 ± 0.11	0.05 ± 0.01	5.80 ± 1.28	0.08 ± 0.08
*Cerebellum*	0.28 ± 0.13	0.18 ± 08	0.10 ± 0.07	0.05 ± 0.01	6.84 ± 1.43	0.10 ± 0.09
*Cingulate gyrus*	0.30 ± 0.15	0.17 ± 0.08	0.16 ± 0.08	0.04 ± 0.01	7.57 ± 1.64	0.12 ± 0.10
*Corpus callosum*	0.10 ± 0.05	0.15 ± 0.07	0.11 ± 0.05	0.02 ± 0.01	3.84 ± 1.26	0.07 ± 0.05
*Hippocampus*	0.22 ± 0.10	0.18 ± 0.10	0.15 ± 0.07	0.03 ± 0.01	6.47 ± 1.20	0.10 ± 0.07
*Insula*	0.24 ± 0.12	0.18 ± 0.08	0.30 ± 0.43	0.04 ± 0.01	6.56 ± 1.20	0.10 ± 0.08
*Occipital cortex*	0.30 ± 0.15	0.19 ± 0.20	0.20 ± 0.08	0.05 ± 0.1	6.67 ± 1.22	0.11 ± 0.09
*Orbitofrontal cortex*	0.28 ± 0.13	0.20 ± 0.08	0.29 ± 0.33	0.05 ± 0.01	6.72 ± 1.25	0.09 ± 0.08
*Globus pallidus*	0.18 ± 0.09	0.21 ± 0.16	0.17 ± 0.09	0.03 ± 0.01	5.39 ± 1.17	0.09 ± 0.06
*Parietal cortex*	0.27 ± 0.13	0.16 ± 0.07	0.19 ± 0.10	0.05 ± 0.01	6.65 ± 1.19	0.09 ± 0.08
*Putamen*	0.28 ± 0.11	0.17 ± 0.09	0.17 ± 0.08	0.05 ± 0.01	6.89 ± 1.44	0.09 ± 0.08
*Temporal cortex*	0.25 ± 0.10	0.19 ± 0.09	0.24 ± 0.20	0.04 ± 0.01	6.63 ± 1.25	0.11 ± 0.09
*Thalamus*	0.26 ± 0.12	0.18 ± 0.07	0.16 ± 0.08	0.04 ± 0.01	6.94 ± 1.50	0.10 ± 0.08
*Whole brain white matter*	0.16 ± 0.06	0.16 ± 0.05	0.15 ± 0.06	0.04 ± 0.01	4.90 ± 0.95	0.07 ± 0.06
*Whole brain grey matter*	0.26 ± 0.12	0.18 ± 0.08	0.17 ± 0.08	0.05 ± 0.01	6.40 ± 1.20	0.10 ± 0.08

### Assessment of test-retest variability

**Fig. 5:**
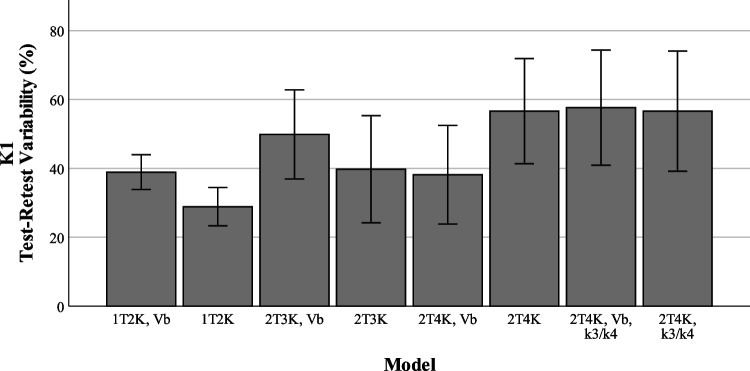
*K*_1_ test-retest variability

**Fig. 6:**
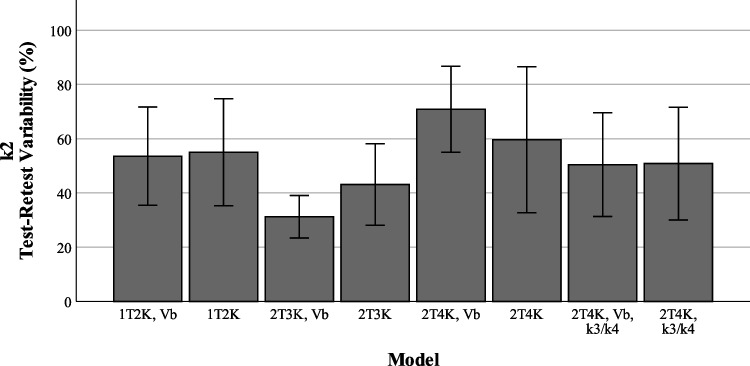
*K*_2_ test-retest variability

**Fig. 7:**
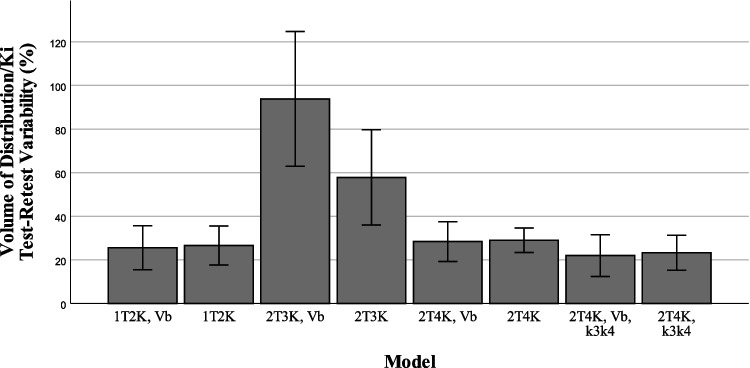
Volume of distribution test-retest variability

Figures 5, 6, and 7 show the reproducibility of the outcome parameters (*V*_*T*_, *K*_*i*_, *K*_1_, *k*_2_) derived from [^18^F]MC225 data using the whole brain grey matter as region of interest for each model. The highest reproducibility between test and retest scans was found when using the reversible two tissue compartment model with fixed *k*_3_/*k*_4_ (TRTV − *V*_*T*_ = 21 ± 21%), the lowest reproducibility when using the irreversible two tissue compartment model with fixed *V*_*B*_ parameter (TRTV − *K*_*i*_ = 93 ± 69%). TRTV of the preferred model based on the AIC scores (2T4K with an estimated *V*_*B*_) was 28 ± 20% for *V*_*T*_, 37.4 ± 22.4% for *K*_1_, and 108.1 ± 69.0% for *k*_2_.

## Discussion

In this study, several compartment models were investigated to select the optimal model for describing [^18^F]MC225 kinetics in humans based on accuracy, precision, and biological assumptions. The present study shows that the 2T4K model with *V*_*B*_ as model parameter is the most accurate model to describe [^18^F]MC225 kinetics in the human brain, and therefore it is the optimal model for quantifying P-gp function at the BBB. Previous preclinical [^18^F]MC225 studies in both rats and non-human primates also have shown that the reversible two tissue compartment model provides the best fit to the PET data, at least for longer scan durations [20, 24]. As [^18^F]MC225 is a substrate of P-gp instead of a tracer that binds to a receptor, the tracer will not follow the same rules of pharmacokinetic quantification as receptor-bound tracers. Therefore, absolute quantification of [^18^F]MC225 is relatively complicated. Based on a biological approach, it could be assumed that *k*_2_ would be the parameter of choice to quantify P-gp function, as it represents the transport from the first tissue compartment back to plasma. On the other hand, Lubberink and colleagues showed that, in general, *K*_1_ is the preferred parameter to describe P-gp function [25]. This could be explained by the fact that pharmacokinetic models are an oversimplified mathematical description of the complex underlying biological processes. If transport by P-gp results in tracer efflux before the tracer actually enters the brain, changes in P-gp function will be reflected in changes in *K*_1_ rather than in *k*_2_. However, *K*_1_ may depend on perfusion and requires a correction for changes in regional cerebral blood flow (CBF) with a separate CBF measurement. In the current study, in addition to the ‘standard’ reversible and irreversible models, a reversible model with a fixed *k*_3_/*k*_4_ parameter was evaluated, to explore the assumption of a stable binding (*k*_3_/*k*_4_) across patients.

The present results show a relatively high standard error for both *K*_1_ and *k*_2_, resulting in low precision for these parameters. Moreover, since *K*_1_ is the product of flow and extraction fraction, changes in *K*_1_ may be due to changes in CBF. In contrast to the high standard errors for *K*_1_ and *k*_2_, the volume of distribution showed a low standard error and therefore, it was selected as the optimal parameter for evaluating BBB P-gp function. The volume of distribution is defined as the ratio of the radioligand concentration in target tissue to that in plasma at equilibrium. This ratio is inversely related to P-gp function.

In cerebral PET studies, the input function needs to be corrected for labelled plasma metabolites, as plasma metabolites usually do not cross the BBB. In the present study, [^18^F]MC225 was relatively stable over time with a parent fraction of more than 60% after 60-min scan duration. For comparison, the widely used P-gp tracer *(R)*-[^11^C]verapamil showed a parent fraction of 45% after 60 min [16]. This would imply that the signal measured with [^18^F]MC225 is less affected by the formation of radio-metabolites, improving the accuracy of P-gp measurements in vivo.

[^18^F]MC225 is a weak P-gp substrate and was developed to measure both increases and decreases in P-gp function. [^18^F]MC225 *V*_*T*_ for the whole brain grey matter region was 6.59, which is substantially higher than the 0.78 reported for *(R)*-[^11^C]verapamil [26]. This is in line with the fact that verapamil is a more avid P-gp substrate than MC225. In addition, in preclinical studies and in a head-to-head comparison with *(R)*-[^11^C]verapamil, higher baseline uptake was found for [^18^F]MC225 [18, 20]. This higher baseline uptake enables measurements of increased P-gp function, which give rise to a reduction in uptake (and *V*_*T*_), as already has been shown in preclinical studies using the P-gp inducer MC111 [27]. Future human studies in, for example, a patient population suffering from treatment-resistant epilepsy are needed to assess the ability of [^18^F]MC225 to measure increases in human P-gp function as well.

One important finding of the present study is the relatively large TRT variability of 28% for *V*_*T*_, which is higher than the 9% TRT variability reported for *(R)*-[^11^C]verapamil [28]. For [^18^F]MC225, TRT variability may be affected by physiological fluctuations of P-gp function and expression, environmental factors, and technical and methodological factors. One other explanation for the large TRT variability is that the lower reproducibility of [^18^F]MC225 might be a result of [^18^F]MC225 being a weaker P-gp substrate, which is transported more gradually across the BBB and, therefore, is more affected by subtle changes in P-gp function [29]. Physiological small changes in P-gp function are present during the day due to the circadian cycle and hormonal influences and although patients were scanned at the exact same time of the day, minor changes in P-gp function could have contributed to the lower reproducibility of [^18^F]MC225 [30]. Furthermore, interindividual outcomes were highly variable (S5-7) and one of the subjects showed an increase in whole brain grey matter *V*_*T*_ of 88%. When excluding this subject from the analysis, the TRT variability decreased from 28.3 ± 20.4 to 20.2 ± 10.1%, which is similar to the test-retest variability of 19% that was previously found in a preclinical [^18^F]MC225 study under controlled conditions [31]. When excluding this subject from the analysis, the TRT variability decreased from 28.3 ± 20.4 to 20.2 ± 10.1%, which is similar to the test-retest variability of 19% that was previously found observed in a preclinical [^18^F]MC225 study under controlled conditions (32). In this preclinical study, the high TRT variability was attributed to the use of isoflurane anaesthesia. However, in the current study, no anaesthesia was used and the high TRT variability can therefore not be explained by these pharmaceutical effects. When comparing the results of [^18^F]MC225 with the current ‘gold standard’ *(R)*-[^11^C]verapamil, it should be noted that in the study of van Assema and colleagues in which a TRV of 9% was found for *(R)*-[^11^C]verapamil, patients were scanned twice on the same day [28]. In the current study, scanning of both test and retest scans on the same day was not possible due to the longer half-life of the radionuclide. There was a minimum interval of 2 weeks between scans, possibly contributing to the higher TRT variability.

Although TRT variability of [^18^F]MC225 *V*_*T*_ in humans is relatively high, under pathophysiological conditions, changes in P-gp function usually are larger, including a nearly 50% reduction in P-gp activity in mild Alzheimer’s disease and a 40% increase in P-gp protein levels in patients with epilepsy [32, 33]. In an ongoing study in which differences in brain [^18^F]MC225 uptake at baseline and after inhibition of the P-gp function are assessed, a difference of 25% was found in the first study subject. Although the high TRT variability may raise concerns about the interpretation of quantitative PET data in individual patients, it does most likely not affect the outcome of this study at group level, as in that case changes are expected to be unidirectional [34] When taken these findings into account, pathophysiological changes in P-gp function might still be measurable using [^18^F]MC225, at least at a group level. Further studies in neurological and psychiatric patient populations are needed to assess whether these changes in P-gp function are large enough (compared with normal subjects) to be measured with [^18^F]MC225.

In the current study, both male and female subjects in the age group 50–80 years were included to ensure that the study population could serve as a representative control group for future studies in Alzheimer’s disease and Parkinson’s disease. Although the main reason for including both males and females was to obtain a representative control group for future studies, this inclusion also allowed for an assessment of possible gender differences in P-gp function at the BBB. However, in contrast to previous literature in which gender differences in P-gp function were present, in this study, no differences in P-gp function were found between male (*V*_*T*_ = 6.6 ± 1.1) and female (*V*_*T*_ = 6.2 ± 1.3) subjects (*p* = 0.595) [35, 36]. This might be due to the relatively small study population of 14 subjects. Future studies are necessary to study the effect of age with [^18^F]MC225. Toyohara et al. stated in the first clinical study with [^18^F]MC225 that P-gp function at the BBB decreases with increasing age. In our study, only a very low correlation (*R*^2^ = 1.25*10^−4^) was found between *V*_*T*_ values of the whole brain grey matter and the increasing age of the study subjects (supplementary data S9). This might be explained by the inclusion of only elderly subjects for which the variability is not as large as in other studies in which ageing and P-gp function was studied.

### Clinical implications and future perspectives

In the present study, compartment modelling was used to study P-gp function at the BBB. However, compartment modelling requires continuous arterial blood sampling, which complicates its clinical implementation. Ideally, for future clinical studies, simplifications of scan procedures are needed. As there is no apparent brain tissue devoid of P-gp transporters and changes in P-gp function will most likely affect the *K*_1_/*k*_2_ ratio, it is not possible to use (traditional) reference tissue models. Therefore, the focus should be on alternative ways to determine the input function, including the use of an image derived input function, which will be facilitated by the introduction of large axial field of view PET scanners.

## Conclusion

[^18^F]MC225 *V*_*T*_, derived using a reversible two-tissue compartment model, is the preferred parameter to describe P-gp function at the human BBB. This outcome parameter has an average test–retest variability of 28%. Although this TRTV is relatively high, changes in P-gp function in pathophysiological conditions are even more extensive and adequate measurements of those alterations in future clinical studies should be feasible with [^18^F]MC225.

### Supplementary Information

Below is the link to the electronic supplementary material.

Supplementary file1 (DOCX 336 KB)

## Data Availability

The data that support the findings of this study are available from the corresponding author, upon reasonable request.

## Abbreviations

1T2K: 1-Tissue compartment model
2T4K: 2-Tissue compartment model reversible
2T3K: 2-Tissue compartment model irreversible
ABC-transporter: ATP-ase binding cassette transporter
AIC: Akaike information criteria
ATP: Adenosine triphosphate
BBB: Blood-brain barrier
CBF: Cerebral blood flow
MRI: Magnetic resonance imaging
NHP: Non-human primate
P-gp: P-glycoprotein/permeability glycoprotein
PET: Positron emission tomography
TLC: Thin layer chromotography
TRV: Test-retest variability
*V*_*B*_: Blood volume
