# Supplementary material for: Quantification of P-glycoprotein function at the human blood-brain barrier using [18F]MC225 and PET
Source: Eur J Nucl Med Mol Imaging. 2023 Aug 8;50(13):3917–27. doi: 10.1007/s00259-023-06363-5 (PMC10611838; doi:10.1007/s00259-023-06363-5)
Supplement: Supplementary file 1 — Supplementary file1 (DOCX 336 KB) [file 259_2023_6363_MOESM1_ESM.docx]

**Supplementary data**

| **Model** | **Input** | **Outcome Parameters** |
| --- | --- | --- |
| 1T2K, V_B_ | One tissue compartment model  Estimated blood volume parameter | V_T_ K1, k2 |
| 1T2K | One tissue compartment model  Fixed blood volume parameter | V_T_ K1, k2 |
| 2T3K, V_B_ | Irreversible two tissue compartment model  Estimated blood volume parameter | K_i_ K1, k2, k3 |
| 2T3K | Irreversible two tissue compartment model  Fixed blood volume parameter | K_i_ K1, k2, k3 |
| 2T4K, V_B_ | Reversible two tissue compartment model  Estimated blood volume parameter | V_T_ BP_ND_ K1, k2, k3, k4 |
| 2T4K | Reversible two tissue compartment model  Fixed blood volume parameter | V_T_ BP_ND_ K1, k2, k3, k4 |
| 2T4K, k3k4 | Reversible two tissue compartment model  Fixed blood volume parameter Fixed k3/k4 | V_T_ K1, k2 |
| 2T4K, V_B_, k3k4 | Reversible two tissue compartment model  Estimated blood volume parameter Fixed k3/k4 | V_T_ K1, k2 |

* Fixated blood volume parameter at 5% for grey matter regions and 3% for white matter regions **Supplementary data S1 – Explanation of the models, input functions and outcome parameters**

| **Subject** | **Scan interval (weeks)** |  | **Administered dose (MBq)*** | **Molar activity (GBq/mmol)** | **Administered cold compound MC225 (μg)** |
| --- | --- | --- | --- | --- | --- |
| **1** | 7 | Test | 96.39 | >128000 | 0.32 |
|  |  | Retest | 233.00 | 733340 | 0.13 |
| **2** | 8 | Test | 163.84 | >200000 | 0.35 |
|  |  | Retest | 163.30 | 358000 | 0.19 |
| **3** | 3 | Test | 230.50 | >400000 | 0.24 |
|  |  | Retest | 230.80 | >500000 | 0.19 |
| **4** | 2 | Test | 228.06 | 1630872 | 0.06 |
|  |  | Retest | 221.00 | 1000000 | 0.09 |
| **5** | 2 | Test | 262.90 | >200000 | 0.56 |
|  |  | Retest | 188.00 | 163119 | 0.49 |

*Mass [^18^F]MC225 <0.27 mg/L

**Supplementary data S2** – [^18^F]MC225 Scan Characteristics


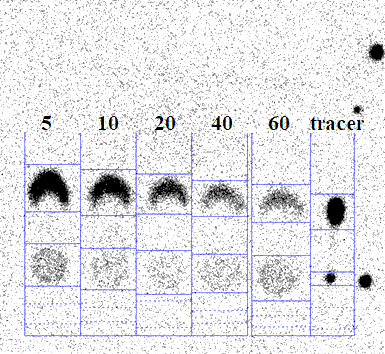


**Supplementary data S3** – Representative example of the TLC plate of one of the study subjects


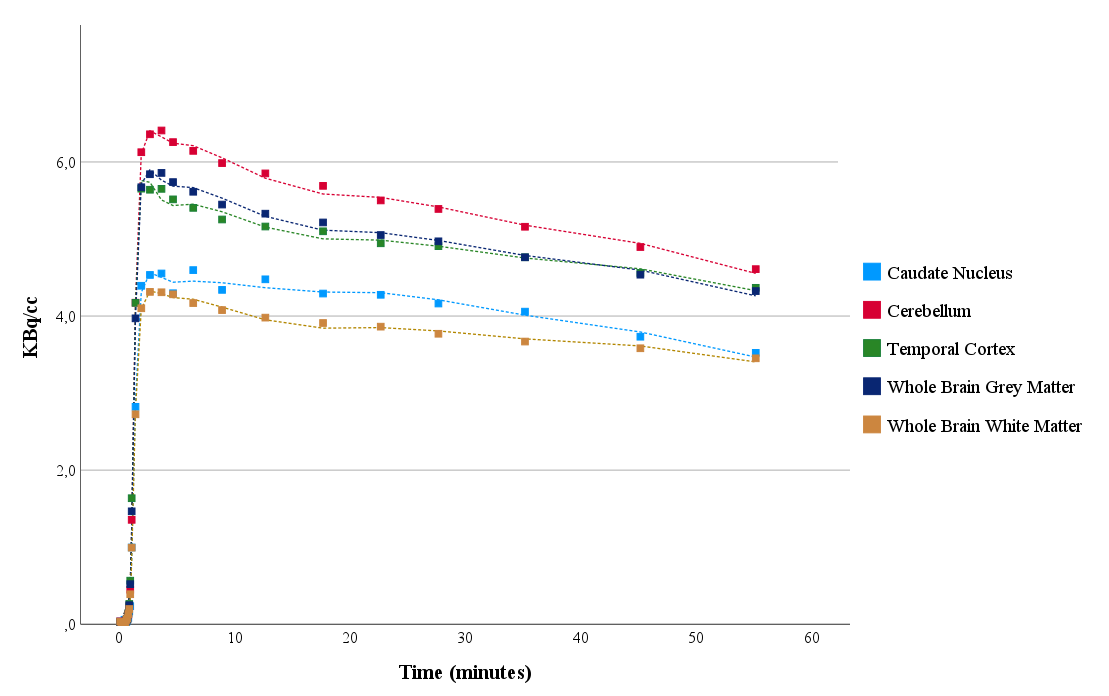


**Supplementary data S4** – Model fits 2T4K model for multiple preselected brain regions of one representative study subject


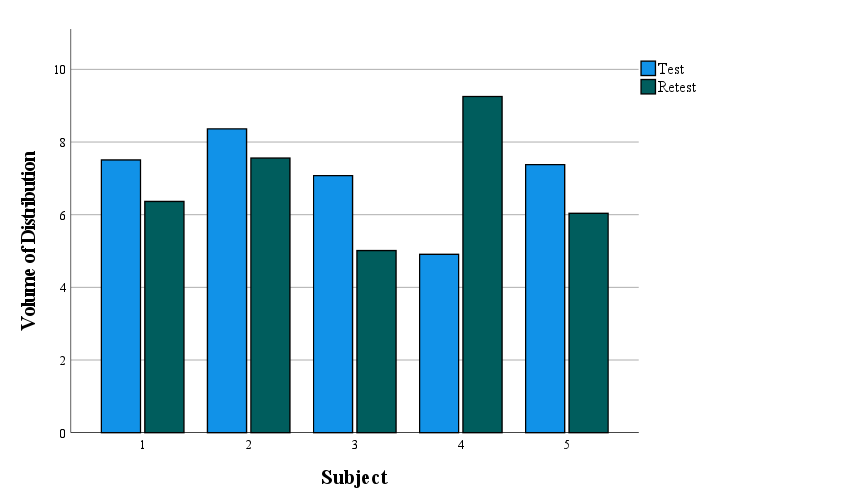


**Supplementary data S5** – V_T_ Test-Retest outcomes of the 2T4K model per subject


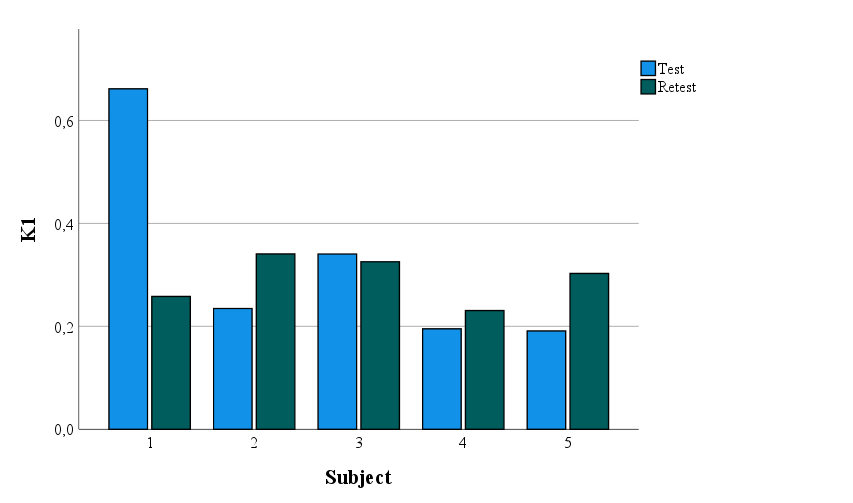


**Supplementary data S6** – K1 Test-Retest outcomes of the 2T4K model per subject


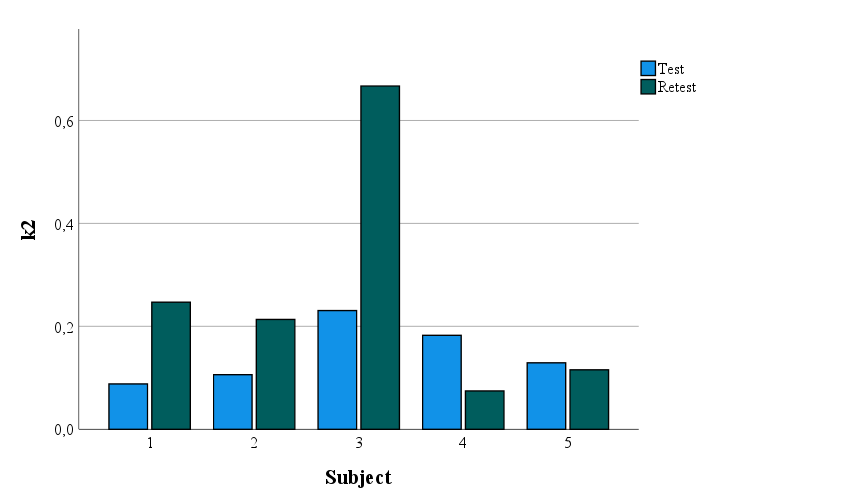


**Supplementary data S7** – K2 Test-Retest outcomes of the 2T4K model per subject


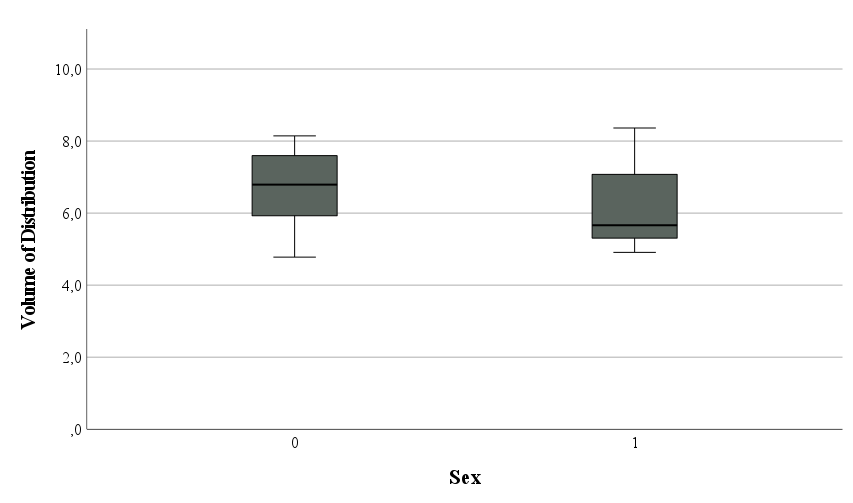


**Supplementary data S8** – Sex differences


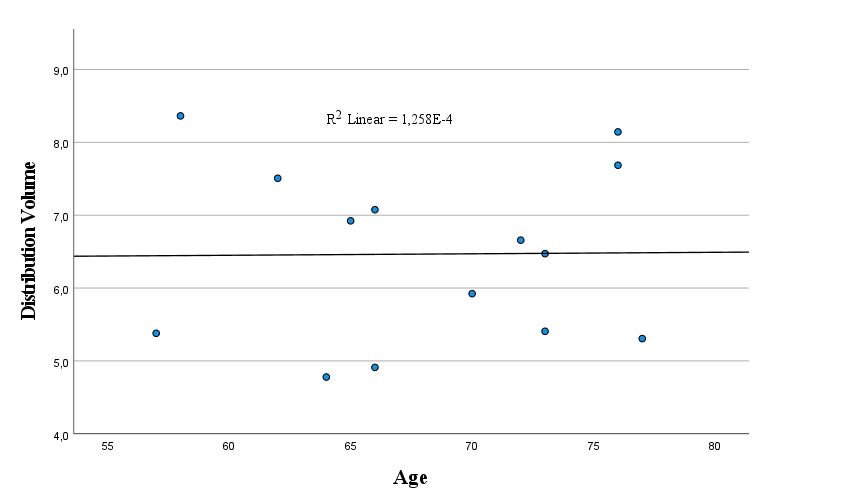


**Supplementary data S9** – Correlation between age and [^18^F]MC225 tracer distribution
